# Case Report: Perioperative nursing management in a patient with dilated cardiomyopathy undergoing EV-ICD implantation

**DOI:** 10.3389/fcvm.2026.1865748

**Published:** 2026-08-26

**Authors:** Shanhu Deng, Hejun Jiang, Lingzhi Huang, Xiaokun He, Luyao Liu, Jiuhong Li, Yurong Wang

**Affiliations:** 1 Xiangya School of Nursing, Central South University, Changsha, China; 2 The Second XiangYa Hospital of Central South University Interventional Operating Room, Changsha, China; 3 The Second XiangYa Hospital of Central South University Nursing Department, Changsha, China; 4 The Second XiangYa Hospital of Central South University Cardiovascular Surgery Department, Changsha, China; 5 The Second XiangYa Hospital of Central South University Cardiology Department, Changsha, China

**Keywords:** case report, dilated cardiomyopathy, EV-ICD, extravascular implantable cardioverter defibrillator, perioperative nursing

## Abstract

This study provides a comprehensive overview of the perioperative nursing management for a patient with dilated cardiomyopathy who underwent extravascular implantable cardioverter-defibrillator (EV-ICD) implantation. Key preoperative nursing interventions included the formation of a multidisciplinary team to assess surgical indications and the patient's overall condition, the enhancement of nutritional support, meticulous preoperative skin preparation, psychological support, thorough equipment inspection, and training for positional cooperation. During the intraoperative phase, rigorous aseptic techniques were employed; nurses collaborated precisely in fluoroscopic and patient positioning, maintained vigilance against mediastinal tissue injury, and adhered to standardized nursing protocols during defibrillation threshold testing. Postoperative nursing care prioritized the prevention and management of mediastinal infections, the prevention of lead displacement, and the management of inappropriate shocks. Intensive monitoring of vital signs and device functionality was conducted, supplemented by comprehensive health education throughout the process and long-term follow-up care. Following standardized treatment and comprehensive nursing care, the patient was transferred to a local hospital in stable condition 19 days post-surgery. A six-month follow-up indicated normal performance of the EV-ICD, with no complications related to the device or the procedure.

## 1 Introduction

Dilated cardiomyopathy (DCM) is a diverse myocardial disorder characterized by the dilation of the left ventricle or both ventricles, coupled with systolic dysfunction, occurring in the absence of abnormal loading conditions or significant coronary artery disease sufficient to induce ventricular remodeling (1). Clinically, DCM predominantly presents as congestive heart failure and various arrhythmias (2, 3). As one of the intractable cardiovascular diseases, DCM is associated with a poor prognosis, with a 5-year survival rate below 50% (4), and approximately 33.3% of patients experience sudden cardiac death (SCD) (5).

Currently, the implantation of an implantable cardioverter defibrillator (ICD) is the cornerstone of clinical strategies for preventing SCD due to malignant arrhythmias (6). With ongoing advancements in medical technology, various types of ICDs have been developed, including the transvenous implantable cardioverter defibrillator (TV-ICD), subcutaneous implantable cardioverter defibrillator (S-ICD), and extravascular implantable cardioverter defibrillator (EV-ICD) (7).

The EV-ICD has been introduced on a global scale, integrating the advantages of both TV-ICD and S-ICD systems to provide a novel therapeutic option for the prevention of sudden cardiac death (8), particularly in patients with DCM (9). However, there is a paucity of globally published studies on perioperative nursing for EV-ICD implantation, leading to a lack of comprehensive clinical experience and standardized guidelines for reference.

In September 2025, our hospital performed an EV-ICD implantation on a patient diagnosed with DCM. Following an extensive multidisciplinary treatment and nursing care protocol, the patient was discharged after a 19-day hospitalization and exhibited stable health during a subsequent 6-month follow-up period. To date, we have successfully conducted this procedure in over ten consecutive cases without encountering any perioperative complications. This paper aims to elucidate the nursing experiences associated with this particular case.

## 2 Clinical information

### 2.1 Brief medical history

A 48-year-old male patient was admitted to the Department of Cardiovascular Medicine on September 10, 2025, with a 6-year history of recurrent exertional dyspnea that had worsened over the preceding month. He had poorly controlled grade 2 hypertension at very high cardiovascular risk, complicated by chronic renal insufficiency and hyperuricemia; an acute gout attack occurred during hospitalization. Cardiac dilatation was first detected 6 years earlier. Physical exertion frequently precipitated acute decompensated heart failure, manifesting as paroxysmal nocturnal dyspnea and bilateral lower-extremity pitting edema; these episodes had led to multiple previous hospitalizations for heart failure exacerbation. There was no documented family history of cardiomyopathy, sudden cardiac death, or inherited myocardial disorders. Persistent chest tightness and dyspnea caused severe fear of sudden cardiac death, leading to marked preoperative anxiety and poor sleep quality. Long-term oral guideline-directed medical therapy for heart failure had failed to achieve sustained symptom control.

Admission vital signs were as follows: body temperature, 36.3 °C; heart rate, 104 beats per min; respiratory rate, 20 breaths per min; blood pressure, 110/84 mmHg. The patient was alert and oriented to person, place, and time. Auscultation of the lungs revealed clear breath sounds without rales or rhonchi. Cardiac auscultation demonstrated a regular rhythm with no significant murmurs over all valvular areas. The abdomen was soft and nontender without rebound tenderness, and mild bilateral lower-extremity pitting edema was noted. New York Heart Association (NYHA) functional class III was assigned based on exercise intolerance and symptoms at rest.

A comprehensive diagnostic workup was completed on admission. Twenty-four-hour ambulatory electrocardiography showed sinus rhythm with frequent atrial premature contractions, paroxysmal atrial tachycardia, multifocal ventricular premature contractions, and five episodes of non-sustained ventricular tachycardia, accompanied by segmental ST-T wave depression and reduced heart rate variability. These findings conferred a substantially elevated risk of malignant arrhythmia and sudden cardiac death. Transthoracic echocardiography demonstrated global cardiac dilatation with diffuse dyssynchronous left ventricular wall motion and a ventricular aneurysm involving the basal inferior-posterior wall of the left ventricle. Moderate mitral regurgitation, mild aortic and tricuspid regurgitation, and dilatation of the ascending aorta and pulmonary artery were identified. Left ventricular ejection fraction (LVEF) ranged from 20% to 27%, with a left ventricular end-diastolic diameter of 88 mm. Cardiac magnetic resonance imaging confirmed an LVEF of 20% and mid-wall late gadolinium enhancement in the left ventricular myocardium; these findings were considered compatible with dilated cardiomyopathy. Laboratory testing revealed a peak N-terminal pro-B-type natriuretic peptide concentration of 20,356 pg/mL and elevated high-sensitivity troponin T, together with increased inflammatory biomarkers including C-reactive protein, interleukin-6, and procalcitonin. Serum creatinine was 301.0 μmol/L, indicating impaired renal function, with concomitant hypoalbuminemia and hyperuricemia. Recurrent hyponatremia and mild metabolic acidosis were observed during the inpatient stay. Portable chest radiography showed marked cardiomegaly and faint bilateral pulmonary opacities suggestive of interstitial infiltrates.

After excluding hypertensive heart disease and rheumatic valvular heart disease, the patient was diagnosed with dilated cardiomyopathy. Given the severely reduced LVEF and recurrent ventricular tachycardia, the patient met the criteria for primary prevention ICD implantation. Unlike S-ICD, EV-ICD provides anti-tachycardia pacing, cardioversion, and post-defibrillation backup pacing. In the setting of severe cardiac dilatation and severely reduced LVEF, the patient was considered to be at high risk of post-defibrillation asystole or bradycardia, making post-shock pacing essential. Therefore, EV-ICD was selected as the optimal device choice. In addition, local medical insurance coverage reduced the patient's financial burden and improved access to the procedure.

### 2.2 Treatment and regression

Inpatient management was immediately initiated with standardized heart failure therapy incorporating diuretics, inotropic agents, and ventricular rate-lowering medications. Device implantation was initially scheduled to mitigate arrhythmic mortality risk.

On hospital day 5, the patient developed fever peaking at 38.5 °C associated with suprapubic discomfort; urinalysis confirmed a urinary tract infection, and empiric meropenem was administered for antibacterial coverage. Dual sets of blood cultures yielded positive growth on September 16, with microbial speciation identifying Staphylococcus lugdunensis. Given a documented penicillin hypersensitivity, clinical pharmacy consultation recommended adjunctive teicoplanin for combination antimicrobial therapy, and the scheduled ICD procedure was deferred to prioritize source control of systemic bacteremia. The patient developed a febrile peak of 39.5 °C with rigors and chills on September 17. Following nine consecutive days of targeted dual-antibiotic therapy, body temperature normalized and serial inflammatory biomarkers demonstrated consistent downward trends.

EV-ICD implantation was performed under general anesthesia on hospital day 14. A retrosternal surgical tunnel was created for extravascular positioning of the defibrillation lead, and defibrillation threshold testing yielded adequate therapeutic energy delivery; the intraoperative course was uncomplicated. Postoperatively, recurrent fever developed alongside a single episode of transient non-sustained ventricular tachycardia. Repeat chest radiography showed progression of bilateral pulmonary infiltrates. After diagnostic evaluation ruled out generator pocket infection and infective endocarditis, combination meropenem and teicoplanin therapy was reinstituted. Aggressive dual antimicrobial treatment, antiarrhythmic intervention, and meticulous volume status regulation resulted in sustained fever resolution, marked amelioration of exertional and resting dyspnea, and complete resolution of bilateral lower-extremity edema.

On hospital day 19, the patient reported substantial relief of dyspnea with stable vital signs. Serial inflammatory markers remained mildly elevated, yet the patient and his legal caregivers repeatedly requested transfer to a local community hospital for prolonged rehabilitation. After comprehensive counseling detailing long-term risks including recurrent systemic infection, device malfunction, and recurrent malignant ventricular arrhythmia, systematic discharge education was completed, and hospital discharge was authorized.

Outpatient follow-up evaluations were conducted at 3 and 6 months post-discharge. Serial EV-ICD device interrogations demonstrated stable electrical parameters throughout surveillance, with no procedural complications encountered, including lead dislodgement, pocket infection, mediastinal tissue injury, or inappropriate shock delivery. The patient exhibited substantial improvements in functional exercise capacity, with near-complete resolution of the severe preoperative anxiety. No recurrent episodes of malignant ventricular arrhythmia were documented across both follow-up time points.

## 3 Discussion

The substernal implantation approach of EV-ICD carries anatomical risks distinct from those of TV-ICD and S-ICD. Its leads are anchored only to mediastinal soft tissue without intracardiac or intravascular fixation, making them susceptible to traction displacement caused by upper limb movement, body position changes, and severe coughing. The pulse generator is embedded within fascial tissue with limited blood perfusion; blind subcutaneous tunneling may retain hematoma and contaminants, thereby increasing the risk of pocket infection. In addition, the substernal operative corridor lies adjacent to vital mediastinal structures, posing a risk of intraoperative tissue injury (10). Data from a global three-year multicenter pivotal trial further quantified these complications: at 3 years, the cumulative incidence of lead dislocation was 2.8%, inappropriate shocks increased from 9.8% at 1 year to 17.5% at 3 years, and pocket or incision infection occurred in 2.5%. Rare adverse events, including pneumothorax, mediastinal hematoma, and lead fracture, have been closely associated with tunnel creation, postoperative compression, and long-term tissue friction (10, 11).

Current mainstream perioperative nursing guidelines are primarily developed for transvenous and subcutaneous ICDs (12, 13). However, standardized nursing protocols tailored to the retrosternal anatomical features of EV-ICD implantation remain lacking, particularly for high-risk patients complicated by preoperative bacteremia and severe hypoalbuminemia (10, 14). To address this gap, this study formulated targeted nursing interventions based on the anatomical characteristics of EV-ICD and developed an integrated nursing pathway covering preoperative risk screening, intraoperative fluoroscopic coordination, and long-term postoperative management. Compared with general ICD nursing consensus (12, 14), this pathway adds targeted risk prevention modules and may serve as a clinical reference for perioperative care of similar high-risk patients.

### 3.1 Focusing on the characteristics of fascia layer implantation, dynamic monitoring, and prevention of mediastinal infection and structural injury

#### 3.1.1 Preoperative preventive care

**Figure 1:**
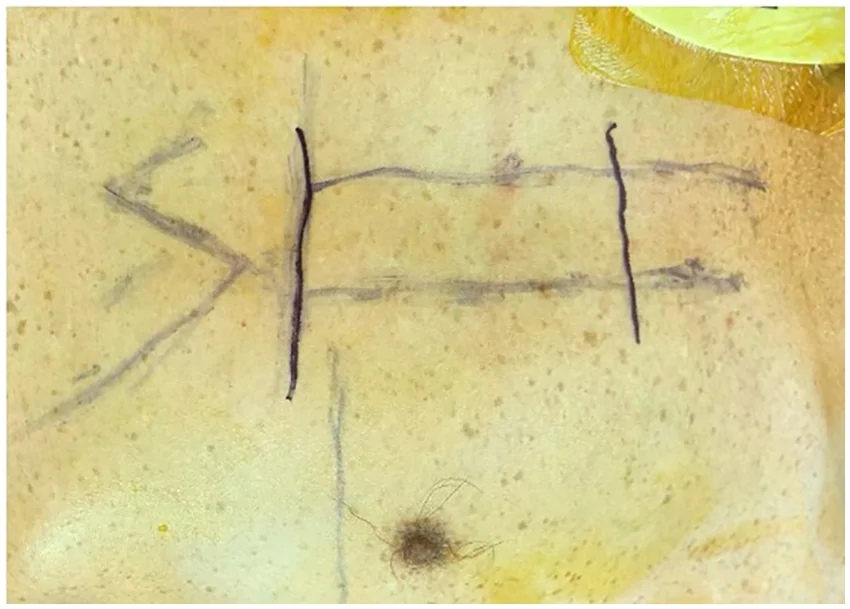
Surgical marking of the implantation site.

**Figure 2:**
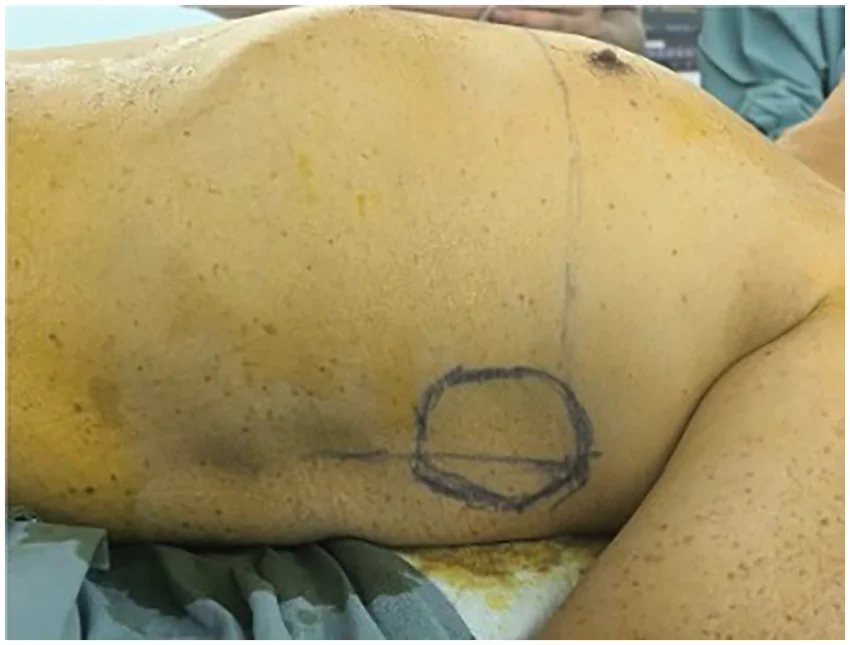
Marking of the pocket position.

Conventional preoperative skin preparation for S-ICD typically covers only limited chest regions (12). In this case, we designed an expanded disinfection area extending from the bilateral shoulders and neck down to the umbilicus, and from the right anterior axillary line to the entire left upper limb. Combined with disposable electric razor shaving and two-stage cleansing using soapy water followed by 2% chlorhexidine alcohol, this protocol effectively reduces intraoperative contamination risk; this measure is not included in generic ICD infection control guidance. Nutritional risk stratification represents another supplementary component of the nursing protocol. The patient's NRS2002 score was 4, indicating high malnutrition risk. A targeted low-salt, high-protein dietary intervention was initiated preoperatively to increase serum albumin and improve anti-infective capacity. This intervention addresses the poor blood supply of EV-ICD fascial pockets, a risk factor that has been overlooked in routine TV-ICD/S-ICD nursing pathways. Prophylactic meropenem (500 mg) was administered intravenously 1 h preoperatively. Preoperative markings were made on the sternum and the lateral pocket site, located in the left 4th to 6th intercostal space along the midaxillary line, as illustrated in Figures 1, 2.

#### 3.1.2 Intraoperative nursing

Nurses in the catheterization laboratory play a crucial role in both the execution and oversight of surgical team preparation and infection control measures. They are responsible for supervising hand hygiene practices among all operating personnel, ensuring the sterility of surgical instruments and dressings, and assisting in the disinfection and draping of the surgical field (12).

#### 3.1.3 Postoperative monitoring and interventions

For routine ICD recipients, postoperative wound observation is usually limited to 7 days. In this study, daily systematic assessments of incision erythema, tenderness, and exudation were scheduled to continue for up to 14 days postoperatively. However, because the patient was discharged on postoperative day 5 for personal reasons, in-hospital assessments were completed until discharge, and subsequent evaluations were conducted through scheduled outpatient or telephone follow-up. This prolonged monitoring period was designed to address the dual infectious risks associated with residual preoperative bacteremia and poorly perfused fascial generator pockets, a high-risk scenario not specifically addressed in previous clinical guidelines. Immediately after the procedure, a sandbag was applied for local compression for 6 h. Subsequently, a compression bandage allowing one-finger clearance was applied to balance hemostasis and local tissue perfusion, thereby avoiding tissue ischemia that may result from the rigid full compression used for transvenous ICDs. Nurses continuously monitored for typical signs of pneumothorax and mediastinal hematoma, including chest pain, dysphagia, and substernal foreign body sensation (15). The patient was instructed to avoid forceful coughing, which may increase retrosternal pressure and lead traction; this restriction is unique to retrosternal devices and is not included in general ICD activity recommendations. Within the first three postoperative days, body temperature was measured four times daily and serial inflammatory markers were monitored to facilitate early detection of occult infection, complementing the postoperative surveillance framework recommended by device infection guidelines (14). The patient was also advised to wear loose clothing and avoid heavy lifting or excessive abduction of the left upper limb to reduce friction at the fascial pocket and the risk of secondary infection (12).

### 3.2 Optimizing intraoperative fluoroscopic collaboration and standardizing positional management

The EV-ICD is implanted within the substernal space. Due to the distinct anatomical characteristics of this region and the limited comprehension of its anatomy within the field of electrophysiology, intraoperative fluoroscopy is essential for accurately delineating sternal morphology, assessing the dimensions of the retrosternal space, and understanding its proximal relationship to mediastinal structures. This imaging technique is also crucial for the precise determination of the device implantation site (7). Consequently, effective positional management and collaboration under fluoroscopic guidance are of paramount importance during the surgical procedure.

#### 3.2.1 Preoperative preparation and collaboration

Preoperative consultations were conducted to elucidate the purpose of intraoperative fluoroscopy, highlight the critical aspects of multi-position projection collaboration, and emphasize the significance of position stabilization to the patient, thereby mitigating anxiety. The patient was instructed to practice maintaining a supine position and immobilizing the left upper extremity to facilitate intraoperative cooperation. The patient's NPO (nil per os) status was rigorously verified for those undergoing general anesthesia, ensuring adherence to a fasting period of 6–8 h for solids and 2 h for clear liquids, with precise documentation of the last intake time. The functionality and imaging clarity of the catheter laboratory fluoroscopy system were assessed. Auxiliary equipment, such as positioning pads and restraints, was prepared. The operational status of EV-ICD implantation instruments and external defibrillators was confirmed. The model and specifications of the EV-ICD generator and leads were verified in conjunction with the operator. Temporary pacemakers and emergency medications, including isoprenaline and atropine, were prepared, and defibrillation pads were pre-applied to ensure readiness for emergencies.

#### 3.2.2 Intraoperative positioning and fluoroscopic coordination

The EV-ICD pocket is strategically positioned between the 4th and 6th intercostal spaces along the left midaxillary line. To prevent interference with intraoperative fluoroscopy, the nursing team collaborated with the anesthesiologist to perform right internal jugular central venous catheterization. Following the induction of general anesthesia, the patient was positioned supine with the shoulder elevated to ensure optimal exposure of the substernal space. The patient's back was aligned closely with the operating table, and the left arm was abducted at an angle of 30°–45° on a movable armrest. ECG electrodes were positioned away from the fluoroscopic field to avoid image obstruction during creation of the substernal tunnel. Because EV-ICD implantation requires continuous anteroposterior and lateral fluoroscopy of the sternum and retrosternal space, any ECG electrodes or cables placed on the anterior chest or sternal region would generate high-density metallic artifacts on fluoroscopy. These artifacts may obscure the guidewire and the anatomical planes of the tunnel, thereby increasing the risk of inadvertent injury to mediastinal structures. Therefore, standard limb leads were used in this case, which allowed adequate ECG signal acquisition without interfering with intraoperative fluoroscopic imaging. Skin protection measures were implemented during limb restraint, and care was taken to avoid spinal twisting to prevent distortion of imaging. Vital signs were meticulously monitored throughout the fluoroscopic procedure. The fluoroscopic angle was promptly adjusted in collaboration with the operator to maintain clear anteroposterior and lateral views, facilitating the confirmation of the guidewire path and precise lead implantation. Consistent patient positioning was maintained throughout the procedure to avert lead deviation due to malposition. The initial lead position was meticulously documented and communicated to the ward nurses, providing a reference for postoperative lead position assessment.

### 3.3 Enhancing positional and activity management for accurate monitoring and prevention of lead dislocation

#### 3.3.1 Postoperative position control

Eight hours following surgery, patients were assisted in mobilizing out of bed. While in the supine position, the head of the bed was elevated to no more than 30°. The affected limb was immobilized, permitting only fist-clenching exercises. Movements involving lifting, abduction, and rotation were prohibited. Specifically, elevating the elbow above shoulder level or raising the upper limb beyond 90° was strictly forbidden (13).

During nighttime rest, a forearm restraint band was utilized to prevent inadvertent limb movements that could exert traction on the lead. Patients were instructed to ensure synchronous movement of the trunk and the affected upper limb during turning to prevent torsion of the trunk. When transitioning out of bed, patients were advised to first sit up and then gradually stand to avoid sudden postural changes that might induce lead traction.

#### 3.3.2 Phased activity guidance

A structured rehabilitation protocol was developed to facilitate staged recovery. Within the first 24 h following surgery, patients were guided through passive exercises, including finger flexion and extension, as well as wrist joint movements, on the operated limb. By 48 h postoperatively, patients were gradually permitted to engage in mild, limited activities involving the limb on the side of the surgical pocket, with slight arm lifting allowed. However, weight-bearing and excessive shoulder movements, particularly during activities such as dressing and combing, were restricted. Upon discharge, patients received instructions to begin gradual, limited shoulder joint activities starting one week after surgery and to progressively resume routine upper limb exercises by one month postoperatively, while avoiding strenuous exercises. It complements general activity-guidance recommendations for EV-ICD recipients.

#### 3.3.3 Dynamic monitoring and evaluation

Continuous electrocardiographic monitoring was conducted for a duration of 72 h post-surgery to assess alterations in pacing signals and sensing thresholds. Symptoms such as chest pain, inadequate sensing, or palpitations were meticulously monitored to provide early detection of potential lead dislocation. A chest x-ray, including anteroposterior and lateral views, was evaluated at 48 h postoperatively and prior to discharge to compare the lead position with immediate postoperative imaging. Clinical manifestations, such as incision traction sensation, muscle twitching, and abnormal device shock alerts, were systematically and dynamically assessed.

### 3.4 Multidimensional strategies for the prevention of inappropriate shocks and the optimization of emergency management and psychological intervention

#### 3.4.1 Preoperative assessment and preventive measures

A thorough preoperative assessment was conducted to evaluate the severity of heart failure, the frequency of arrhythmic episodes, and exercise tolerance, utilizing electrocardiography and 24-h Holter monitoring. Preoperative psychological nursing represents a vital intervention, with catheterization laboratory nurses playing a pivotal role in its execution (16, 17). The Generalized Anxiety Disorder-7 (GAD-7) scale was employed to assess the patient's psychological condition, yielding a score of 17, which signifies severe anxiety. The primary nurse engaged in daily communication with the patient, elucidating the operational principles, antitachycardia pacing function, and shock-related aspects of the EV-ICD through the use of a physical model. Additionally, the nurse shared successful case studies of similar patients. Emotional support from family members was also encouraged to mitigate the patient's apprehension regarding the surgery and potential shocks.

#### 3.4.2 Intraoperative device programming and testing

Intraoperative monitoring of electrosurgical unit utilization was conducted throughout the procedure to ensure that the probe did not come into contact with the metallic housing of the implanted pulse generator, thereby preventing device dysfunction due to electromagnetic interference (18). Certified EV-ICD technicians were responsible for device testing and programming. Following the placement of the substernal defibrillation electrode at the designated target site, the following parameters were assessed: R-wave amplitude ranging from 3.3 to 4.0 mV, pacing threshold at 4 V with a pulse width of 2.0 ms, low-voltage impedance between 337 and 601 Ω, and high-voltage impedance at 78 Ω, all of which conformed to the surgical requirements.

#### 3.4.3 Postoperative monitoring and mitigation of interference

Continuous electrocardiographic monitoring was conducted for 72 h following surgery. The ICD event log was systematically recorded to promptly identify factors that could trigger inappropriate shocks, such as myoelectric interference and electromagnetic signals. Electrocardiographic changes and device programming data were closely monitored in real-time. The patient and their family members received guidance on strategies to prevent electromagnetic interference. It was determined that common household appliances, including refrigerators, microwave ovens, and mobile phones, did not compromise device functionality. However, contact with equipment that generates direct vibrations or electromagnetic waves, such as electric massage beds, induction cookers, and electric drills, should be avoided, as should areas with strong magnetic fields, such as those near large motors and substations. It is imperative that medical personnel are informed of the EV-ICD implantation prior to any medical examinations, and specific programming adjustments are necessary before undergoing MRI procedures (14).

#### 3.4.4 Long-term health education and patient follow-up

Patients and their families were educated on the normalcy of experiencing mild chest vibrations and palpitations during EV-ICD operation to mitigate undue anxiety stemming from unfamiliar physical sensations. They were instructed on how to accurately identify abnormal symptoms, such as palpitations, chest tightness, chest pain, dizziness, shortness of breath, and excessively rapid, slow, or irregular pulse. Emergency contact information was provided, and the significance of seeking prompt medical consultation, as opposed to passive observation, was underscored. Education on the recognition of infections was enhanced. Patients and their families were instructed to promptly contact medical personnel upon the occurrence of symptoms such as fever, redness, swelling, warmth, pain, or exudation at the incision site (19). Emphasis was placed on wound care precautions: dressings must remain clean and dry, and changes to wound dressings should only be conducted by professional surgical staff. Direct manual contact with the incision or the application of self-medication was strictly prohibited. An EV-ICD identification card was issued, detailing the device model, implantation date, and physician contact information. A standardized follow-up schedule was established, with device interrogation and echocardiography scheduled at 1, 3, and 6 months postoperatively, followed by regular follow-ups every 6–12 months, and provisions for unscheduled visits in case of discomfort. Contact numbers for the department and QR codes for online consultations were provided. Regular follow-ups via telephone and WeChat were conducted to monitor rehabilitation progress and device performance, address inquiries, and provide personalized health guidance.

## 4 Outcomes and evaluation

The patient exhibited grade A incision healing following surgery. On postoperative day three, the NRS pain score was recorded at 2, with complete pain relief achieved by a week post-surgery. The patient's total hospital stays lasted 19 days, during which no perioperative complications were observed, including mediastinal hematoma, pneumothorax, mediastinal infection, lead dislocation, or pocket infection. At the three-month follow-up, the parameters of the EV-ICD remained stable, demonstrating normal anti-tachycardia pacing function, and no malignant arrhythmias were detected. Chest radiography confirmed the lead position remained unchanged. The self-designed postoperative health-knowledge questionnaire for EV-ICD patients consists of 20 items with a total score of 100 points, covering five dimensions: medical insurance-related cognition, device awareness, posture safety, infection prevention and control, as well as electromagnetic protection and follow-up. Questionnaire assessment revealed that the patient's preoperative knowledge score was 35 points, which increased to 90 points at discharge. Additionally, the GAD-7 score decreased from 17 preoperatively to 3 at discharge, indicating mild anxiety and a significant improvement in psychological status. To date, our team has successfully completed perioperative nursing care for 10 patients undergoing EV-ICD implantation, with no nursing-related complications reported (This statement only describes the institutional clinical background experience and shall not be regarded as evidence supporting the conclusions of the present case report.).

## 5 Conclusion

In the case of a patient with dilated cardiomyopathy who underwent EV-ICD implantation, perioperative nursing strategies were specifically developed in accordance with the anatomical nuances of the retrosternal approach. Proactive nursing interventions were employed to mitigate potential complications, including mediastinal structural injury, mediastinal infection, lead dislocation, and inappropriate shocks. Cardiac function management was intensified, taking into account the underlying heart failure associated with dilated cardiomyopathy. Emphasizing the distinctive functional attributes of the EV-ICD, targeted efficacy assessments and standardized health education initiatives were also conducted. This study on nursing practice encapsulates the essential perioperative nursing considerations for the implantation of an EV-ICD in patients with dilated cardiomyopathy, serving as a reference for the clinical management of analogous cases. Future efforts should focus on refining nursing protocols, which includes the creation of a standardized health education manual for EV-ICD recipients and the implementation of a continuous nursing system utilizing remote monitoring. These advancements aim to enhance the nursing experience and rehabilitation outcomes for patients.

## 6 Limitations

This study has several limitations. It is a single-center, single-case observational study without a control group. The nursing protocols proposed here require validation in large-sample, prospective multicenter studies before broad clinical application. The six-month follow-up duration precluded observation of late-onset complications reported in three-year pivotal trials, such as chronic fascial inflammation and long-term inappropriate shock events. In addition, the patient's early transfer to a local hospital resulted in incomplete inpatient rehabilitation data, limiting comprehensive analysis of medium-term wound healing. Furthermore, owing to the limited sample size, the self-designed postoperative health-knowledge questionnaire for EV-ICD patients used in this study has not undergone reliability and validity testing, which constrains its generalizability and standardized application. Future multicenter studies enrolling larger numbers of high-risk EV-ICD patients with infectious complications are needed to refine and validate the nursing strategies summarized in this report.

## 7 Patient perspective

According to the patient, “Upon first learning that I needed implantation of the novel retrosternal EV-ICD, I knew little about this new technology and worried about uncertain therapeutic effects and elevated surgical risks. While awake during the procedure, I experienced obvious pulling sensations in the chest and substernal distending discomfort. In the early postoperative period, I had persistent substernal dull pain, chest compression and a foreign-body sensation. I dared not take deep breaths or cough normally for fear of tugging the internal leads and damaging the surgical wound. Meanwhile, I was greatly concerned about high costs of surgical consumables and unclear medical-insurance reimbursement ratios, fearing a heavy personal financial burden. I also worried about the need for re-operation for future device replacement and recurring expenses for subsequent follow-up visits and device interrogations. After clinical staff systematically explained the technical advantages of EV-ICD, the normality of postoperative physical discomfort, postoperative precautions, medical-insurance reimbursement policies and the long-term device replacement cycle, my concerns regarding surgery and recovery were substantially alleviated, and my health-knowledge level improved markedly. Although I had mastered self-care skills before discharge, I still harboured certain worries about long-term device performance and persistent life-style restrictions and hoped to receive long-term follow-up guidance.”

## Data Availability

The original contributions presented in the study are included in the article/Supplementary Material, further inquiries can be directed to the corresponding author.
